# Efficacy of Three-Dimensional Bioactive Composites in Long Bone Repair with Photobiomodulation

**DOI:** 10.3390/ma18081704

**Published:** 2025-04-09

**Authors:** Sebastião Júlio Rodrigues Júnior, Letícia Carlucci dos Santos, Daniela Vieira Buchaim, Marco Antonio Hungaro Duarte, Murilo Priori Alcalde, Benedito Barraviera, Rui Seabra Ferreira Júnior, Ana Maria de Guzzi Plepis, Virgínia da Conceição Amaro Martins, Paulo Sérgio da Silva Santos, Marcelo Rodrigues da Cunha, Rogerio Leone Buchaim, Karina Torres Pomini

**Affiliations:** 1 Postgraduate Program in Structural and Functional Interactions in Rehabilitation, University of Marilia (UNIMAR), Marilia 17525-902, Brazil; jrsebasport@hotmail.com (S.J.R.J.); leticia.carlucci@facop.com.br (L.C.d.S.); karinatorrespomini@unimar.br (K.T.P.); 2 Dentistry School, Faculty of the Midwest Paulista (FACOP), Piratininga 17499-010, Brazil; 3 Medical School, University Center of Adamantina (FAI), Adamantina 17800-000, Brazil; danibuchaim@alumni.usp.br; 4 Graduate Program in Anatomy of Domestic and Wild Animals, Faculty of Veterinary Medicine and Animal Science, University of São Paulo (FMVZ/USP), São Paulo 05508-270, Brazil; 5 Department of Dentistry, Endodontics and Dental Materials, Bauru School of Dentistry, University of São Paulo (FOB/USP), Bauru 17012-901, Brazil; mhungaro@fob.usp.br (M.A.H.D.); malcalde@fob.usp.br (M.P.A.); 6 Center for the Study of Venoms and Venomous Animals (CEVAP), São Paulo State University (Univ Estadual Paulista, UNESP), Botucatu 18610-307, Brazil; benedito.barraviera@unesp.br (B.B.); rui.seabra@unesp.br (R.S.F.J.); 7 Graduate Program in Tropical Diseases, Botucatu Medical School (FMB), São Paulo State University (Univ Estadual Paulista, UNESP), Botucatu 18618-687, Brazil; 8 São Carlos Institute of Chemistry, University of São Paulo (USP), São Carlos 13566-590, Brazil; amplepis@iqsc.usp.br (A.M.d.G.P.); virginia@iqsc.usp.br (V.d.C.A.M.); 9 Department of Surgery, Stomatology, Pathology and Radiology, Bauru School of Dentistry, University of São Paulo (USP), Bauru 17012-901, Brazil; paulosss@fob.usp.br; 10 Postgraduate Program in Health Sciences, Faculty of Medicine of Jundiaí (FMJ), Jundiaí 13202-550, Brazil; marcelocunha@g.fmj.br; 11 Interunits Graduate Program in Bioengineering (EESC/FMRP/IQSC), University of São Paulo (USP), São Carlos 13566-970, Brazil; 12 Department of Biological Sciences, Bauru School of Dentistry (FOB/USP), University of São Paulo (USP), Bauru 17012-901, Brazil; 13 Medical School, University of Marilia (UNIMAR), Marilia 17525-902, Brazil

**Keywords:** bone substitutes, fibrin sealant, fibrin biopolymer, fibrin, hydroxyapatite, nanohydroxyapatite, low-level laser therapy, photobiomodulation, scaffolds, regenerative medicine

## Abstract

Different treatments have been proposed for morphofunctional bone repair; however, they are not always efficient and have limitations. In this experimental study, we present matrix composites with a possible synergistic effect acting with scaffolds for bone growth and use of photobiomodulation (PBM) to accelerate this tissue repair. Thus, the objective was to evaluate the effect of PBM in the repair of a long bone (tibia) of rats filled with biomimetic collagen matrices with nanohydroxyapatite and heterologous fibrin biopolymer (FB). Forty-eight rats were distributed into eight groups (*n* = six each): Blood Clot (BC), Blood Clot + PBM (BCP), Matrix (M), Matrix + PBM (MP), Fibrin Biopolymer (FB), Fibrin Biopolymer + PBM (FBP), Matrix + FB (MFB), Matrix + FB + PBM (MFBP). A 2.0 mm bone defect was created in the proximal third of the left tibia. The BCP, MP, FBP, and MFBP groups underwent PBM during surgery and maintained twice a week until euthanasia at 42 days. Microcomputed tomography (micro-CT), histomorphological and histomorphometric analyses were performed. Micro-CT analysis revealed that PBM influenced cortical interposition between the remnant and newly formed bone. Histologically, no exacerbated inflammatory focus or foreign body-type granulomatous reaction was observed in any group; however, a vast collagenous matrix with a more oriented and thicker spatial conformation was observed in the PBM-treated groups. Histomorphometrically, the BCP, MP, and MFBP groups showed significantly higher values compared to the other groups. Specifically, the BC group presented a mean bone tissue density of 68.33 ± 7.394, while the BCP and MP groups showed 99.83 ± 11.87 and 99.67 ± 20.58, respectively (*p* < 0.05). Qualitative analysis of collagen fibers indicated enhanced organization and maturation in PBM-treated groups. This study concluded that the association of PBM in the repair of long bones in rats, filled with biomimetic collagen matrices with nanohydroxyapatite and fibrin biopolymer, presented results that contribute to the improvement of bone growth, together with the association of scaffolds.

## 1. Introduction

The structural and functional regeneration of damaged or lost bone tissue is a challenge for regenerative medicine, since bone has limited regenerative capacity and this biological process may fail, leading to delayed consolidation and resulting in a fibrotic union [1,2]. Despite the clear improvements in bone defect reconstruction resulting from tissue engineering and the development of new bone substitutes, these approaches are still insufficient to achieve native morphofunctional restoration [3]. Consequently, the significant impacts on the economy and the patient’s well-being necessitate further research.

Despite advancements, several gaps remain in the field of bone regeneration. Current treatments often fail to fully restore the native structure and function of bone tissue [4]. Many synthetic materials used in bone grafts can cause immune reactions or fail to integrate properly with the host tissue [5]. Additionally, achieving the right balance between mechanical strength and bioactivity in scaffolds remains a significant challenge [6].

To address these gaps, we selected specific scaffolds based on their potential to overcome these limitations. Collagen is the main component of the organic portion of bone tissue, providing a scaffold for cell growth and a low immunogenic response [7]. Nanohydroxyapatite enhances the biomechanical properties of the scaffold, increasing bone resistance to stress and improving cell invasion [5]. Fibrin biopolymer, known for its bioadherence and moldability, mimics the final phase of the coagulation cascade, promoting angiogenesis and tissue remodeling [8].

The combination of collagen, nanohydroxyapatite (nHA), and fibrin in a single scaffold aims to enhance their synergistic properties to optimize bone regeneration. Collagen, the main organic component of the bone matrix, provides a three-dimensional structure that supports cell adhesion and proliferation, while also offering biocompatibility and biodegradability. nHA, which mimics the mineral phase of bone, provides mechanical rigidity and support, promoting osteoconduction and osteoinduction [8,9,10].

Fibrin acts as a natural biopolymer that facilitates hemostasis, cell adhesion, and tissue remodeling. The integration of these components seeks to replicate the hierarchical structure of native bone tissue, creating a favorable microenvironment for bone regeneration. Previous studies have demonstrated that scaffolds composed of collagen and nHA improve mechanical and biological properties compared to pure collagen scaffolds, while the addition of fibrin has shown potential to further enhance bioactivity and tissue integration [8,9,10].

Consequently, given the significant impacts on the economy and the patient’s quality of life, there is a need to develop biomimetic synergistic strategies that incorporate factors intrinsically involved in and determining the formation of new bone tissue [4]. New studies have been conducted to develop three-dimensional matrices, such as natural polymers, which mimic the physiological extracellular matrix, providing a favorable microenvironment for the recruitment and proliferation of osteoprogenitor cells, biologically active molecules, and growth factors in locu [11].

In this context, collagen matrices have been widely analyzed, as they are the main component of the organic portion of the tissue, which gives the bone unique characteristics, as well as the formation of a scaffold for cell growth. Collagen offers several advantages, including low immunogenic response across species, natural abundance, hemostatic promotion, and versatile manipulation [7]. To enhance biomechanical properties and improve bone resistance to stress, particularly in long bones, it is often combined with bone substitutes such as nanohydroxyapatite. This combination also optimizes cell invasion time within the surgical site, contributing to improved scaffold applicability [5].

Other natural hydrogels, such as chitosan, alginate, gelatin, and hyaluronic acid, have also been explored for bone tissue regeneration. Chitosan, for example, is known for its biocompatibility, biodegradability, and antimicrobial properties, but may require chemical modifications to enhance its mechanical strength and stability [12].

Alginate is another promising hydrogel due to its ability to form hydrogels under mild conditions and its biocompatibility; however, the lack of cell adhesion sites can limit its effectiveness unless combined with other materials [13]. Gelatin, derived from collagen, retains many of the beneficial properties of collagen but can be less stable and may require crosslinking to improve its mechanical properties [14]. Hyaluronic acid is valued for its role in promoting cell migration and proliferation, but its rapid degradation in vivo can be a drawback [15].

Despite this, the repair of defects using bone substitutes remains challenging, as the materials need to be manually adapted to the recipient bed during surgery, to provide adequate cell diffusion. The complexity of these procedures can lead to modifications in the properties of the scaffold and consequently create spaces between the graft and the remaining bone [16]. Studies have demonstrated the applicability of heterologous fibrin biopolymer as a biological scaffold that is perfectly moldable to the surgical site and has bioadherence capacity at the graft and remaining bone interface [8,17]. This is possible due to its blood-like constituent elements that mimic the last phase of the coagulation cascade, resulting in a highly adaptable three-dimensional fibrin network. In addition, it is biologically active, which facilitates the adhesion of endothelial cells, fibroblasts, and osteoprogenitors, promoting angiogenesis and tissue remodeling [8].

Fibrin presents several advantageous characteristics, including hemostasis, bioadherence, moldability, and reduced shear stress when combined with biomaterials. Additionally, it is biodegradable and forms a bioactive matrix with cell-binding sites. Consequently, it has garnered significant interest in therapeutic modalities aimed at reconstructing bone defects, driving research into optimal concentrations [18].

Photobiomodulation (PBM) has emerged as a promising method in bone tissue engineering due to its ability to promote cell proliferation, osteogenic differentiation, and bone regeneration. Studies have shown that PBM can enhance the healing process of bone defects by modulating inflammatory responses, stimulating neoangiogenesis, and increasing the production of osteogenic markers such as alkaline phosphatase (ALP) and calcium deposits [19,20]. However, there are still gaps in understanding the optimal parameters for PBM, such as wavelength, energy density, and treatment duration, which need to be addressed to maximize its therapeutic potential [21].

With advances in photobiomodulation therapy (PBM), using low-level lasers, research has demonstrated its use in several medical and dental areas, demonstrating significant and promising results [22]. This is because the electromagnetic radiation of the laser acts on cell photoreceptors, promoting a photochemical interaction capable of inducing an increase in cellular metabolism and consequently different general biological effects such as analgesia, anti-inflammatory and biostimulation [23,24]. Thus, PBM induces earlier, more intense, and more extensive biological responses compared to methods that do not utilize light [25,26,27].

Recent studies conducted by our group of researchers demonstrated speed in the bone repair process in defects filled with the association of fibrin biopolymer composites and biomaterials, when biostimulated with laser radiation, which projects a new biomimetic matrix favorable to tissue growth [22,28].

Therefore, we aim to perform analyses to investigate the bone microarchitecture of tibial defects using a new collagen matrix composite with nanohydroxyapatite and fibrin biopolymer, associated or not with photobiomodulation. This research justifies its applicability and can prospectively be incorporated as scientific and technological advances, to optimize time and reduce the cost of treatment in orthopedic and oral rehabilitation.

## 2. Materials and Methods

### 2.1. Porcine Serosa Collagen Matrix with Nanohydroxyapatite (M)

Anionic collagen was obtained from commercially purchased porcine serosa. The serosa was cleaned with water, cut and washed in baths of 0.5% acetic acid (J.T. Baker^TM^, Phillipsburg, NJ, USA) and 0.5% sodium hydroxide solutions (J.T. Baker^TM^, Phillipsburg, NJ, USA) to remove blood and fat. It was subsequently subjected to alkaline hydrolysis in a solution with hydroxides, chlorides and sulfates of K^+^, Ca^2+^ and Na^+^ for 120 h. This solution of hydroxides and salts was removed, and the serosa was passed through an aqueous solution with the same salts. Subsequently, all salts were removed by washing in solutions of 3% boric acid (J.T. Baker^TM^, Phillipsburg, NJ, USA), deionized water and, 0.3% EDTA (Mallinckrodt, St. Louis, MO, USA), and again with deionized water, pH 6.0. The material was lyophilized and subsequently solubilized in acetic acid (Merck KGaA, Darmstadt, Germany), pH 3.5 to obtain a concentration of 4% by mass.

For the synthesis of nanohydroxyapatite, 100 mL of a 0.01 mol L^−1^ solution of cetrimonium bromide (Sigma-Aldrich, St. Louis, MO, USA) was used and slowly added in 0.6 mol L^−1^ of K_2_HPO_4_. After the addition, the pH was adjusted to 12 with NaOH (Merck KGaA, Darmstadt, Germany) and the mixture was stirred for 2 h. A 1.0 mol L^−1^ solution of CaCl_2_ (Sigma-Aldrich, St. Louis, MO, USA) was prepared and added to the previous solution, under constant stirring. The suspension formed was placed under reflux for 6 h, and subsequently in ultrasound for 1 h. Then, the suspension was washed with deionized water and ethanol. Then, the precipitate was placed at 40 °C for 12 h for solvent evaporation and then calcined at 550 °C for 5 h. The average horizontal diameter of the particles was determined to be 168.6 ± 92.8 nm. The average vertical diameter of the particles was 98.4 ± 33.6 nm [29].

The 4% collagen gel was diluted to 1.5% with a suspension of nanohydroxyapatite in acetic acid (J.T. Baker^TM^, Phillipsburg, NJ, USA), pH 3.5, using the ratio of 30 mg of nanohydroxyapatite for every 10 g of 1.5% collagen gel. From this gel with nanohydroxyapatite, the matrices were made, obtained by lyophilization of approximately 6 g of gel in 11 cm/1.5 cm Teflon^®^ molds. Collagen and nanohydroxyapatite matrices were neutralized in ammonia vapor for a period of 2 h and then aerated under air flow for at least 72 h.

### 2.2. Heterologous Fibrin Biopolymer (FB)

The fibrin biopolymer was kindly provided by the Center for the Study of Venoms and Venomous Animals of the São Paulo State University—UNESP, Botucatu, Brazil (CEVAP), whose components and application formula are in accordance with the patents (registration number: BR 102014011432-7, issued on 8 December 2015 by the National Institute of Industrial Property of Brazil (INPI)) [30]. Initially, it was called fibrin glue or fibrin sealant, but due to its diversity of uses, it is currently called fibrin biopolymer. It was subjected to a phase I/II clinical trial, which demonstrated its safety for therapeutic use in humans, standing out as a promising therapeutic potential [31]. Its formulation is composed of three separate solutions, previously thawed, mixed, and homogenized before application. Component 1 is thrombin-like (gyroxin), obtained from the venom of *Crotalus durissis terrificus*. The diluent comprises synthetic calcium chloride and component 2, fibrinogen (cryoprecipitate) derived from the blood of *Bubalus bubalis* (buffalo) [32,33,34].

### 2.3. Photobiomodulation (PBM) by Low-Level Laser Therapy (LLLT)

Low-intensity laser photobiomodulation treatment was performed using the Therapy XT device (DMC^TM^, São Carlos, Brazil), as shown in Table 1.

### 2.4. Experimental Model

**Figure 1:**
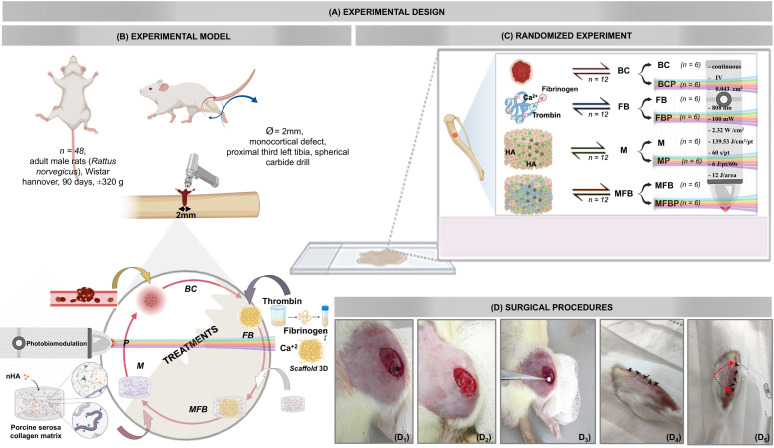
Experimental Protocol and Surgical Procedures for Bone Defect Repair Study. (**A**) Experimental design. (**B**) Random allocation of 48 adult male rats and monocortical drilling of 2.0 mm in diameter with a spherical carbide drill. (**C**) Distribution of animals into groups according to the type of defect filling, and treatment or not by photobiomodulation: Blood Clot (BC), Blood Clot + PBM (BCP), Matrix (M), Matrix + PBM (MP), Fibrin Biopolymer (FB), Fibrin Biopolymer + PBM (FBP), Matrix + FB (MFB), Matrix + FB + PBM (MFBP). (**D**) Surgical procedure, red double broken arrow; (**D_1_**) a 20 mm long linear incision; (**D_2_**) defect in the proximal third of the tibia; (**D_3_**) collagen matrix with nanohydroxyapatite, and collagen matrix associated with fibrin biopolymer, respectively; (**D_4_**) suture; and (**D_5_**) intraoperative photobiomodulation therapy.

Forty-eight adults male Wistar Hannover rats (*Rattus norvegicus*), 90 days old, weighing approximately 320 g, from and kept at the Central Bioterium of the University of Marília (Figure 1A,B), were used. They were cared for according to the guidelines of the Animal Use Ethics Committee (CEUA) of the University of Marília, having approved the study project prior to the beginning of the experiments (Protocol 048/2020 of 19 August 2021). When applicable, this study followed the ARRIVE (Animal Research: Reporting In Vivo Experiments) checklist guidelines. The animals were kept in conventional cages initially containing 4 animals each, with food and water “ad libitum”, in a climate-controlled environment with air conditioning at a temperature of 22 ± 2 °C, air exhaust and a light-dark cycle of 12 h each. After the experimental surgery, the animals were housed in cages individually.

The inclusion criteria for the animals used were male, healthy, and young adults to avoid interference from hormonal factors and ensure adequate metabolic and physiological conditions [35,36]. The sample size was based on bioethical principles of reducing the number of animals, with statistical reliability.

The rats were randomly distributed into 8 groups according to the type of defect filling and treatment by photobiomodulation or not (Figure 1C): Blood Clot Group (BC/*n* = 6): defect filled with blood clot; Blood Clot plus Photobiomodulation Group (BCP/*n* = 6): defect filled with blood clot and photobiomodulation; Matrix Group (M/*n* = 6): defect filled with collagen/nanohydroxyapatite matrix; Matrix plus Photobiomodulation Group (MB/*n* = 6): defect filled with collagen/nanohydroxyapatite matrix and photobiomodulation; Fibrin Biopolymer Group (FB/*n* = 6): defect filled with heterologous fibrin biopolymer; Fibrin Biopolymer plus Photobiomodulation Group (FBP/*n* = 6): defect filled with heterologous fibrin biopolymer and photobiomodulation; Matrix plus Fibrin Biopolymer Group (MFB/*n* = 6): defect filled with collagen/nanohydroxyapatite matrix and heterologous fibrin biopolymer; and Matrix plus Fibrin Biopolymer plus Photobiomodulation Group (MFBP/*n* = 6): defect filled with collagen/nanohydroxyapatite matrix and heterologous fibrin biopolymer and photobiomodulation.

### 2.5. Surgical Procedures

The animals were weighed on a precision scale (MicroNal^TM^ Precision Equipment, São Paulo, Brazil) and anesthetized with an intramuscular injection of tiletamine hydrochloride associated with zolazepam (10 mg/kg—Telazol^TM^; Fort Dodge Laboratories, Fort Dodge, IA, USA) (Figure 1D). Trichotomy was performed with a hair trimmer (Multigroom 3000 MG3711/15, Philips^TM^, Drachten, The Netherlands) on the left pelvic limb in the lateral and dorsal region, and antisepsis of the surgical field was performed with a 10% Polyvinyl Pyrrolidone Iodine PVPI solution (Rioquimica^TM^, São José do Rio Preto, Brazil).

The surgical procedure was performed independently, on a surgical bench, with a change in material for each specimen. The animals were immobilized on the operating table, positioned in the left lateral decubitus position. Next, a 20 mm linear incision was made in the craniocaudal direction with a carbon steel scalpel blade no. 15 (Embramax^TM^, São Paulo, Brazil), reaching deep into the skin and muscle fascia, reaching the periosteum, which was carefully retracted with a syndesmotome.

A 2.0 mm diameter monocortical perforation was made in the proximal third of the left tibia, reaching deep into the bone marrow, without damaging the contralateral cortex, using a No. 6 carbide spherical bur (Microdont^TM^, São Paulo, Brazil) inserted into the contra-angle (Driller^TM^, Carapicuiba, Brazil) together with the electric micromotor (Driller^TM^ BLM 600 Baby, Carapicuiba, Brazil), at low speed (1500 rpm), with saline irrigation, without heating the operated site, thus obtaining a rounded surgical cavity, without spicules, preserving the integrity of the adjacent structures.

Group BC (Blood Clot): In the animals in this group, bone defects were created and left unfilled with biomaterials, allowing the formation of a natural blood clot at the time of trephination. Group M (Collagen/nanohydroxyapatite matrix): Bone defects in the animals in this group were filled exclusively with a matrix composed of collagen and nanohydroxyapatite. Group FB (Fibrin Biopolymer): In this group, the defects were filled with a fibrin biopolymer prepared in a 1:1:1 ratio using 15 µL of gyroxin, 15 µL of diluent and 15 µL of fibrinogen. After complete polymerization, the resulting fibrin matrix was applied directly to the surgical bed. MFB Group (Collagen/nanohydroxyapatite matrix with fibrin biopolymer): For the animals in this group, the collagen/nanohydroxyapatite matrix was incorporated into the fibrin biopolymer in the same 1:1:1 ratio (15 µL of gyroxin, 15 µL of diluent and 15 µL of fibrinogen). This combination was prepared in an Eppendorf tube, where the matrix was mixed with the biopolymer before being applied to the bone defect. After filling the cavities, the periosteum and other tissues were repositioned and sutured. The region was carefully cleaned with gauze moistened with topical antiseptic, 2% chlorhexidine (Riohex^TM^, Rioquímica, São José do Rio Preto, Brazil).

After this step, all experimental groups were subdivided according to whether or not they received photobiomodulation treatment. The animals in the BCP, MP, FBP, and MFBP groups received laser therapy. The treatment began in the immediate postoperative period and continued twice a week until euthanasia (42 days). After the surgical procedures, a single dose of antibiotic (Flotril^TM^ 2.5%, Schering-Plough, Rio de Janeiro, Brazil) was administered intramuscularly at a dose of 0.2 mL/kg, and the analgesic Dipyrone (Analgex V^TM^, Agener União, Sao Paulo, Brazil) at a dose of 0.06 mL/kg, maintained for 3 days, in addition to the continuation of the analgesic acetaminophen (Paracetamol, Medley^TM^, São Paulo, Brazil) at a dose of 200 mg/kg, 6 drops/animal available in the drinking water until the time of euthanasia. Throughout the experiment, the animals’ behavior was monitored, observing any changes that demonstrated pain.

After 42 days of post-surgery, all animals were euthanized in a quiet environment away from other animals, by administering the barbiturate thiopental sodium 2.5% intraperitoneally (IP, 150 mg/kg, Cristália^TM^, Itapira, Brazil), associated with the local anesthetic lidocaine hydrochloride at a dosage of 10 mg/kg).

After confirmation of the animal’s death, the left pelvic limb was removed and the tibia was dissected and disarticulated, preserving the supraperiosteal soft tissues. The collected tissue was fixed in 10% buffered formalin for 48 h.

### 2.6. Sample Size Calculation and Power Analysis

To ensure the robustness and reliability of our study, we performed a power analysis prior to the commencement of the experiments. The power analysis was conducted to determine the minimum sample size required to detect a statistically significant effect of PBM on bone regeneration with a power of 80% and an alpha level of 0.05 [37,38,39,40]. This analysis was based on effect sizes reported in previous studies examining similar interventions.

Specifically, we used the following parameters for our power analysis. Effect Size (Cohen’s d): Based on previous literature, we estimated a medium effect size (d = 0.5) for the impact of PBM on bone regeneration. Significance Level (α): We set the alpha level at 0.05, which is a commonly accepted threshold for statistical significance. Power (1-β): We aimed for a power of 80%, meaning there is an 80% chance of detecting a true effect if it exists. Using these parameters, the power analysis indicated that a minimum of 6 animals per group would be sufficient to achieve the desired statistical power.

### 2.7. Interobserver Reliability for Histomorphometric Measurements

To ensure the accuracy and consistency of our histomorphometric data, we conducted an interobserver reliability assessment. Two independent observers, blinded to the group assignments, performed the measurements. The interobserver reliability was quantified using the intraclass correlation coefficient (ICC), which is a widely accepted statistical method for assessing agreement between observers. The ICC values obtained were above 0.80, indicating excellent reliability and consistency between the observers [41,42].

### 2.8. Control of Potential Confounders

To minimize the impact of confounding variables, we implemented several strategies: Randomization: Animals were randomly assigned to different experimental groups to ensure that potential confounders were evenly distributed across the groups. Blinding: Both the researchers conducting the experiments and those analyzing the data were blinded to the group assignments to reduce bias. Statistical Control: We included potential confounders as covariates in our statistical analyses. This approach helps to adjust for the influence of these variables on the outcomes of interest.

### 2.9. X-Ray Computed Microtomography (Micro-CT)

After fixation of the collected anatomical specimens, they were subjected to an X-ray beam scan on a SkyScan 1174v2 micro-computed tomography (Bruker-micro-CT^TM^, Billerica, MA, USA) at the Bauru School of Dentistry (Endodontics FOB-USP, Bauru, Brazil). The X-ray beam source (Cone-Beam) was operated at 50 kV, 800 uA, using a Cu + Al filter. The specimens were stabilized inside the equipment to prevent any type of movement during scanning. They were then rotated 180°, with a rotation step of 0.5, and isotropic resolution of 19.6 µm, generating an acquisition time of approximately 35 min per specimen.

The images obtained from each specimen were analyzed and reconstructed using NRecon v.1.6.9.8 software (Skyscan^TM^, Kontich, Belgium) in ap-proximately 1000 to 1100 slices according to the anatomical parameters adopted. The software Data Viewer^TM^ v. 1.4.4 64 bits (linear measurements of the coronal, transaxial and sagittal axes) and S^®^ v.2.4.0 r868, (Bruker-micro-CT^TM^, Billerica, MA, USA) were used for two-dimensional and three-dimensional visualization, respectively, and then to perform the qualitative analysis of the newly formed bone tissue.

### 2.10. Histotechnical Processing

After micro-CT, the pieces were placed in running water for 24 h and subjected to demineralization in ethylenediaminetetraacetic acid (EDTA) solution, a solution containing 4.13% tritiplex^TM^ III (Merck KGaA, Hessen, Germany) and 0.44% sodium hydroxide (Labsynth^TM^, São Paulo, Brazil), in which weekly changes were made for a period of approximately 60 days. After complete demineralization, confirmed by dental radiography, the pieces were dehydrated in an increasing series of ethyl alcohol, diaphanized in xylene and embedded in Histosec^TM^ processed paraffin (Merck^TM^, Hessen, Germany), according to the standardized histotechnical protocol in the histology laboratory of the University of Marília (UNIMAR, Marília, Brazil). Subsequently, longitudinal, semi-serial sections (50 µm interval) were performed until reaching the central region of the defect, using a semi-automatic microtome Leica RM2245 (Leica Biosystems^TM^, Wetzlar, Germany). The sections obtained, with a thickness of 5 µm, were subjected to the histological stain’s hematoxylin-eosin (HE), Masson’s trichrome and picrosirius-red.

### 2.11. Qualitative Histomorphological Analysis

For the histomorphological description of the bone defect, images of slides stained with Masson’s trichrome (10× objective) and HE (2× and 40× objectives) were used, considering the entire extension of the defect, to evaluate the pattern of bone repair in all experimental groups. The existence and quality of granulation tissue, immature or mature/lamellar bone, the degree of filling of the newly formed tissue, and inflammatory infiltrate were analyzed in the surgically created defect. Four semi-serial sections of the surgical bed of each defect were evaluated in an Opticam^TM^ O400S light microscope (Opticam Microscopy Technology, Doral, FL, USA), and the photographs were captured in 2×, 10×, and 40× objectives with the attached digital camera (Opticam^TM^ 0.5×, Doral, FL, USA), using the image capture software Opticam Microscopy OPTHD version ×64, 3.76815 (2003–2016, Opticam Microscopy Technology^TM^, Doral, FL, USA), in the histology research laboratory of the University of Marília (UNIMAR, Marília, Brazil).

### 2.12. Quantitative Histomorphometric Analysis

Quantitative (histomorphometric) analysis of the percentage of new bone volume was performed with 2× magnification images, stained in HE, using the point counting planimetry method. For this, a previously established grid with 88 points [43] was superimposed on the image of the defect area of each animal (injured cortical and medullary areas); each point that overlapped the newly formed tissue was considered, and the total density was evaluated by the occupancy in % of the image covering the defect in its entirety (Supplementary Material, Figure S1).

The size of the grid used was 13.2 × 9.6 cm with a spacing of 1.2 cm between each point marked on a transparent sheet. The measurement of the areal densities of the analyzed sections was performed using the equation D = ΣPN/PT × 100, where PN indicates the number of overlapping points in the bone neoformation and PT the total number of points included in the overlapping grid.

To ensure reliability, each measurement was performed in triplicate for each histological section, and the final percentage value (%) was calculated as the average of all analyzed animals.

### 2.13. Birefringence Analysis of the Collagen Fibers of the Bone Defect

Picrosirius-red stained histological slides were evaluated under polarized light to determine the quality and quantity of the newly formed organic matrix throughout the experimental periods of defect repair. Defect images were obtained at 5× and 20× objectives using a Leica DFC 310FX high-resolution digital camera (Leica Microsystems^TM^, Wetzlar, Germany) connected to a Leica DM IRBE confocal laser microscope and LAS 4.0.0 capture system (Leica Microsystems^TM^, Heerbrugg, Switzerland) at the Integrated Research Center (CIP, Bauru School of Dentistry FOB USP, Bauru, Brazil). Two histological fields were analyzed, corresponding to the defect border and the central area, to allow identification and analysis of collagen quality by the birefringence of the organization of the fiber bundles.

### 2.14. Statistical Analysis

The comparative histomorphometric results within each group were submitted to a one-way ANOVA variance test and Tukey’s post-test (Tukey’s multiple comparisons test); significance levels and considerations were determined at 5% (*p* < 0.05). The program used for statistical analysis was GraphPad^TM^ (version Prism 8, La Jolla, CA, USA).

## 3. Results

### 3.1. X-Ray Computed Microtomography (Micro-CT) Analysis

All specimens were scanned after 42 days using computed microtomography, and the images were reconstructed two- and three-dimensionally in the sagittal and transaxial axes to observe the structural details of the bone in the area of the surgically created defect. The scans of each tibia were examined qualitatively, since it was not possible to evaluate the mineral density in the cortical and intramedullary region between the formed bone and the implanted material, since no difference was observed in the threshold levels, revealing a possible underestimation of the bone definition.

**Figure 2:**
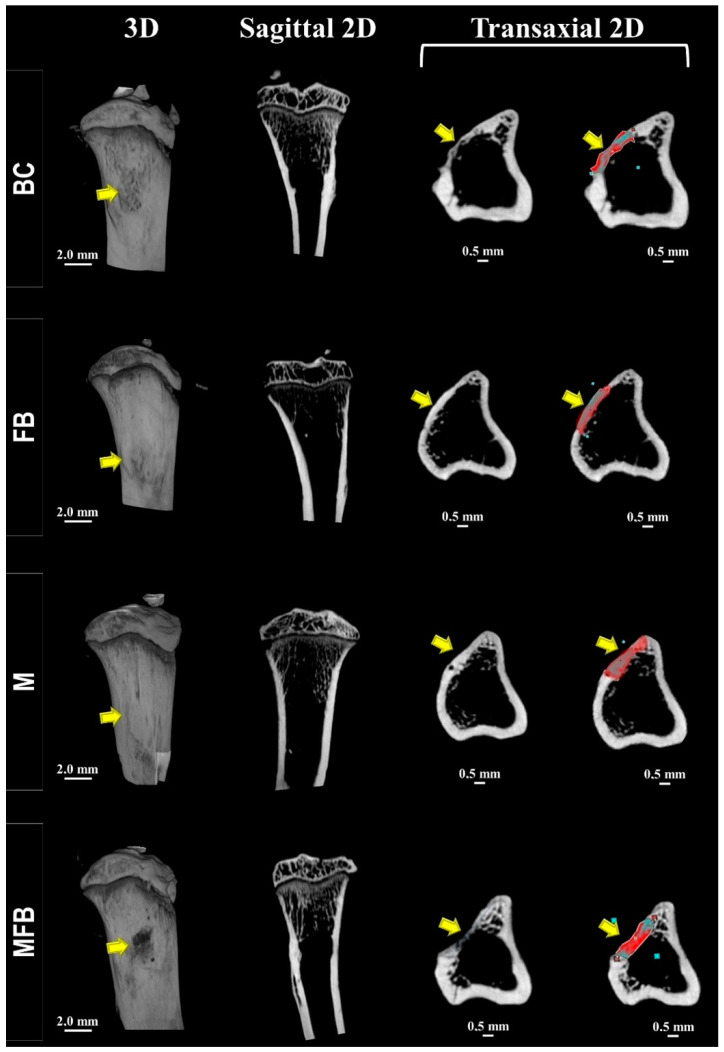
A series of 2D- and 3D-rendered microtomographic images showing the location of the surgical area in the sagittal and transaxial sections in the groups not treated with photobiomodulation at 42 days. Blood Clot (BC), Fibrin Biopolymer (FB), Matrix (M), Matrix + FB (MFB). Representative images of a 2 mm tibial defect with closure of the defect by a thin bone plate (yellow arrow/red zone—cortical lesion). In the transaxial section, the blue-colored region represents an area of lower mineralization compared to the adjacent bone tissue, suggesting potential heterogeneity in bone mineral density. The scale bar is 2 mm for 3D images and 0.5 mm for 2D (transaxial) images.

**Figure 3:**
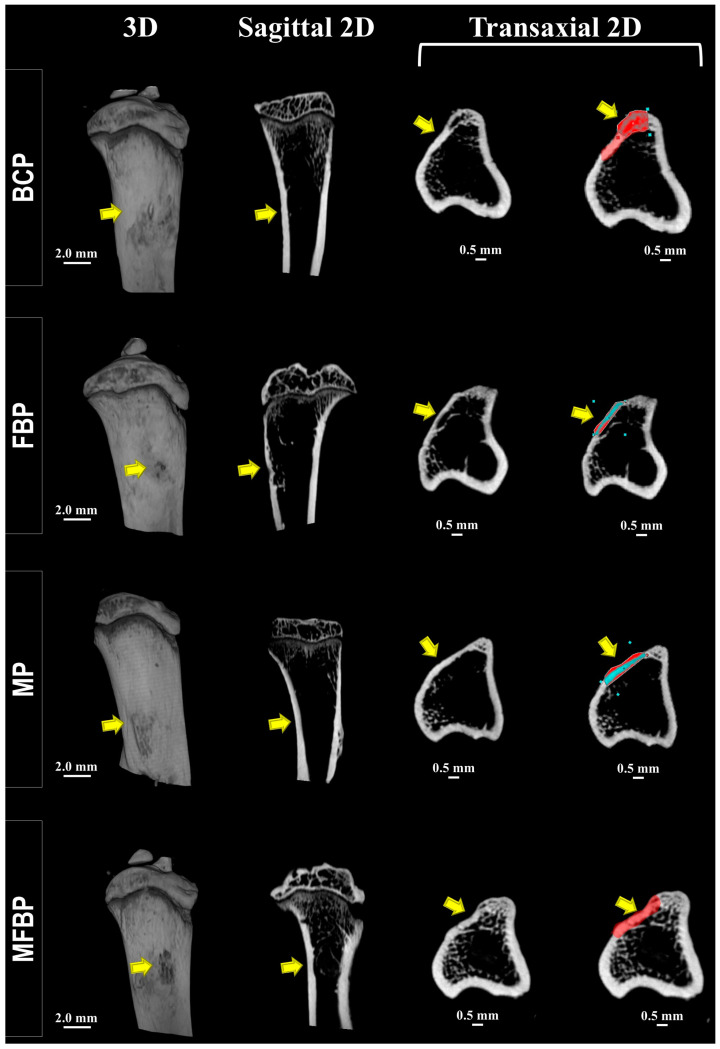
A series of 2D- and 3D-rendered microtomographic images showing the location of the surgical area in sagittal and transaxial sections in the groups treated with photobiomodulation at 42 days. Blood Clot + PBM (BCP), Fibrin Biopolymer + PBM (FBP), Matrix + PBM (MP), Matrix + FB + PBM (MFBP). Representative images of a 2 mm tibial defect with closure of the defects by a thin bone plate (yellow arrow/red zone—cortical lesion). In the transaxial section, the blue-colored region represents an area of lower mineralization compared to the adjacent bone tissue, suggesting potential heterogeneity in bone mineral density. The scale bar is 2 mm for 3D images and 0.5 mm for 2D (transaxial) images.

In all experimental groups, a linear radiopaque region corresponding to the monocortical closure by a thin bone plate was observed, without the complete re-establishment of the native bone thickness. In the underlying medullary area, there was a discrete bone mass at the trabecular limits, which may be correlated with the low density of the new intramedullary bone and the osteoid matrix. The margins of the defect could not be readily identified in the groups treated with photobiomodulation due to complete bone union of the operated site, unlike what was observed in BC, M, FB, and MFB. The presence of nanohydroxyapatite granules could not be distinguished from the entire area of the original defect, both in the cortical plate and in the medullary cavity, due to contiguous bone formation (Figure 2 and Figure 3).

**Figure 4:**
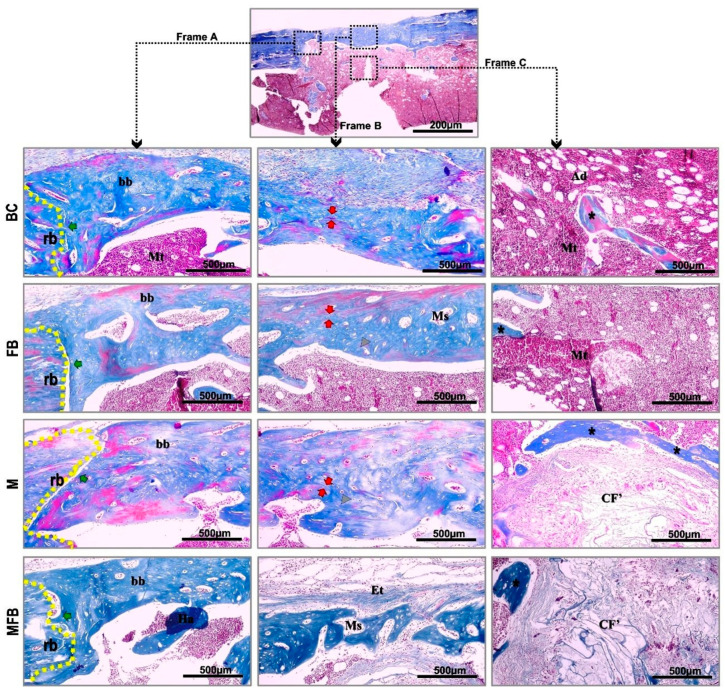
Histological images captured by conventional light microscopy of tibial bone defects, without laser photobiomodulation, in Masson’s trichrome. Frame A—marginal area; Frame B—central area of the bone plate; Frame C—medullary area. Blood Clot (BC), Fibrin Biopolymer (FB), Matrix (M), Matrix + FB (MFB). Abbreviations: b (dashed line)—remaining border, bb—bone bridge, Mt—medullary tissue, Ms—medullary spaces, asterisk—newly formed bone tissue, Et—bone lining epithelium, red arrows—immature tissue transitioning to bone maturation, Ha—histological artifact, CF’—thin and disorganized collagen fibers, green arrow—clear separation of remaining bone and newly formed bone tissue, gray triangle—osteocytes surrounded by their gaps in the osteoid matrix, Ad—adipocytes. 10× image capture lens and 500 scale bar.

**Figure 5:**
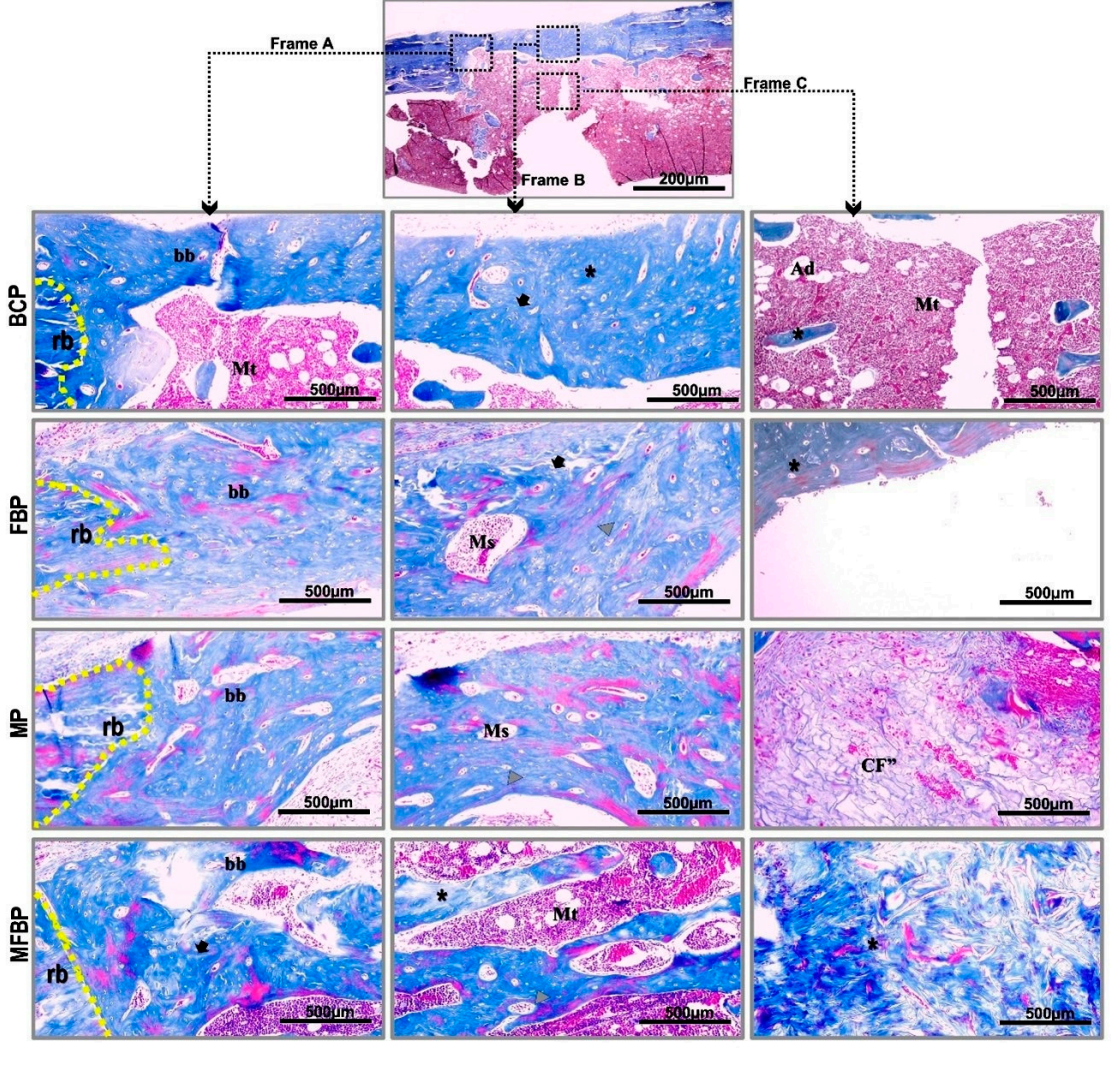
Histological images captured by conventional light microscopy of tibial bone defects, with laser photobiomodulation, in Masson’s trichrome. Frame A—marginal area; Frame B—central area of the bone plate; Frame C—medullary area. Blood Clot + PBM (BCP), Fibrin Biopolymer + PBM (FBP), Matrix + PBM (MP), Matrix + FB + PBM (MFBP). Abbreviations: rb (dashed line)—remaining border, bb—bone bridge, Mt—medullary tissue, Ms—medullary spaces, asterisk—newly formed bone tissue, CF”—thicker collagen fibers, black arrow—lamellar/mature bone tissue. gray triangle—osteocytes surrounded by their lacunae, Ad—adipocytes. 10× image capture lens and 500 scale bar.

The results of the micro-CT analysis indicated that the increase in bone formation was predominantly uniform throughout the tibial defect. However, a higher bone density was observed in the marginal regions of the defect, as evidenced by the histological images (Figure 4 and Figure 5). The histomorphometric analysis corroborated these findings, showing a homogeneous distribution of new bone formation, except for specific areas where the density was significantly higher (*p* < 0.05).

### 3.2. Histomorphological and Histomorphometric Analysis of Newly Formed Bone Tissue

In none of the specimens analyzed was a mononuclear infiltrate clearly identified (by light microscopy at the site of the defect) or even multinucleated giant cells closely associated with the surface of the material, not characterizing a progression of tissue response to chronic inflammation.

All defects demonstrated complete bone union of the peripheral and deep margins at 42 days. However, in the groups treated with photobiomodulation, the new bone revealed a denser lamellar structure in the marginal area of the defect and more sparse osteocytes throughout this surface, with a typical appearance of bone maturation.

The separation of the new bone tissue from the adjacent remnant was conspicuous in the BC, FB, and M groups, except in MFB, with the locus of osteoid matrix transitioning to maturation at the periphery of the defect site, unlike the photobiomodulated groups that presented the entire extension of the lesion intact and without traces of trepanation. Evidence of osseointegration was observed in all defects in groups M, MFB, MP, and MFBP, as the implanted material was completely incorporated into the new cortical tissue, and direct apposition of new bone was observed in the medullary cavity, intertwining it (Figure 4 and Figure 5).

**Figure 6:**
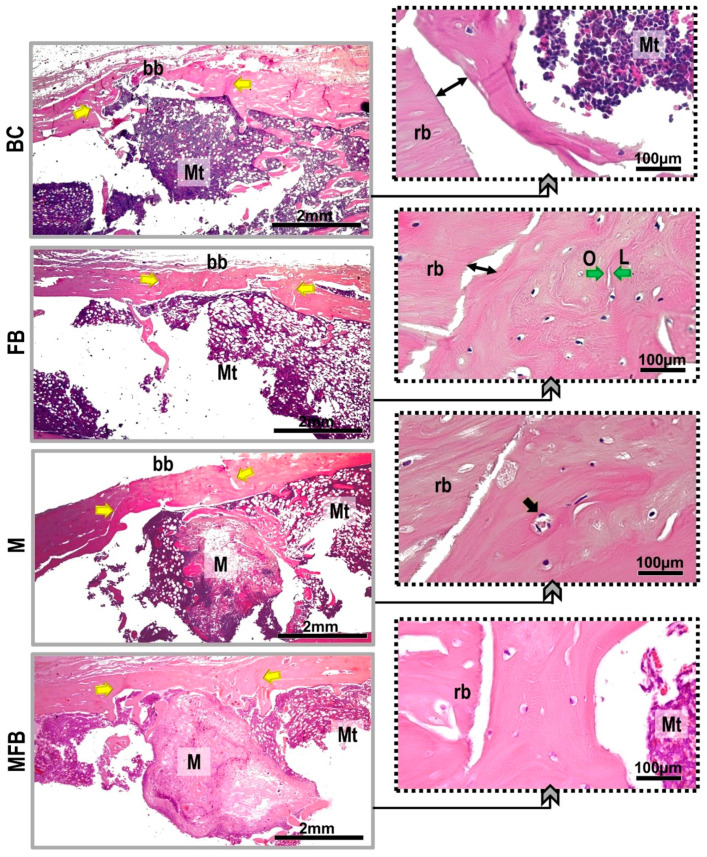
Histological images captured by conventional light microscopy of tibial bone defects, without laser photobiomodulation, HE staining. Blood Clot (BC), Fibrin Biopolymer (FB), Matrix (M), Matrix + FB (MFB). Abbreviations: Mt—medullary tissue, rb—remaining border, bb—bone bridge (yellow arrow), Green arrows—osteoid matrix transition and lamellar bone tissue, Black arrow—osteocytes trapped in lacunae, Double arrow—clear separation of native bone and new bone formation, O—osteoid, L—lamellar bone; M—matrix. The camera was equipped with a 2× objective, and the insets were magnified at 40×. The scale bars are 2 mm and 100 µm, respectively.

**Figure 7:**
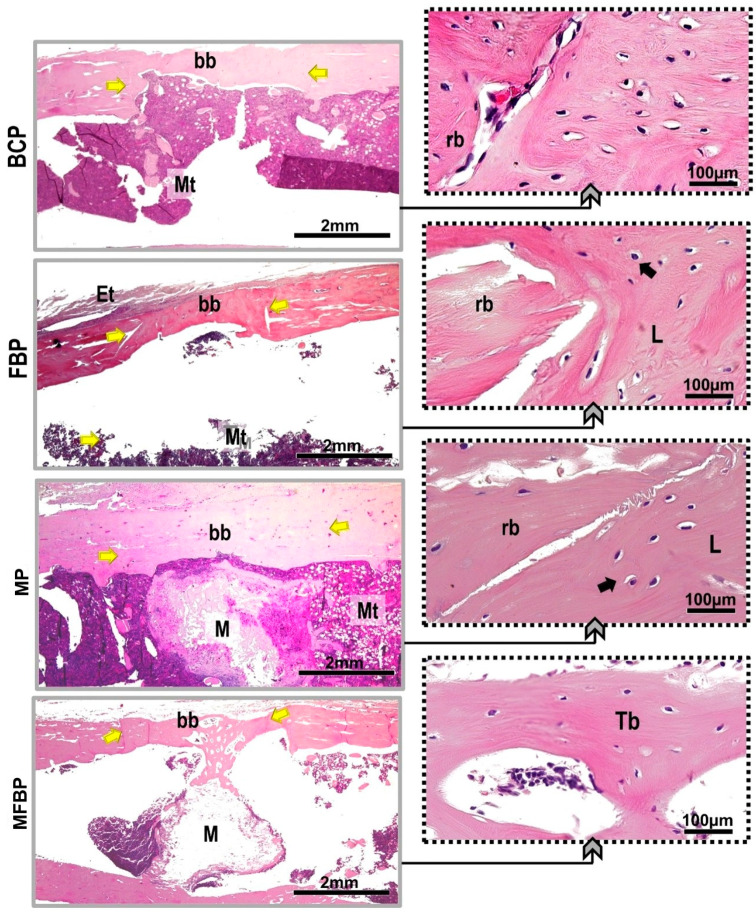
Histological images captured by conventional light microscopy of tibial bone defects, with laser photobiomodulation, stained in HE. Blood Clot + PBM (BCP), Fibrin Biopolymer + PBM (FBP), Matrix + PBM (MP), Matrix + FB + PBM (MFBP). Abbreviations: Mt—medullary tissue, bb—remaining border, bb—bone bridge (yellow arrow), Black arrow—osteocytes trapped in lacunae, Et—bone lining epithelium, L—lamellar cap; Tb—bone trabeculae, M—matrix. The camera was equipped with a 2× objective, and the insets were magnified at 40×. The scale bars are 2 mm and 100 µm, respectively.

All experimental groups confirmed monocortical linear closure in hematoxylin and eosin staining, but the dimensioning of the new bone formed was not restored when compared to the native bone. In the groups treated with photobiomodulation (BCP, MP, FBP, and MFBP), the formation of bone bridges, predominantly cortical, was observed in the remodeling process, being thicker in relation to the groups not treated with photobiomodulation (BC, M, FB, and MFB), transitioning to a height equivalent to the remnant. In the underlying medullary area, a clear amount of red bone marrow was shown. In the groups with bone formation induced by grafting, the presence of the material was still present, with surrounding trabeculae (Figure 6 and Figure 7).

**Figure 8:**
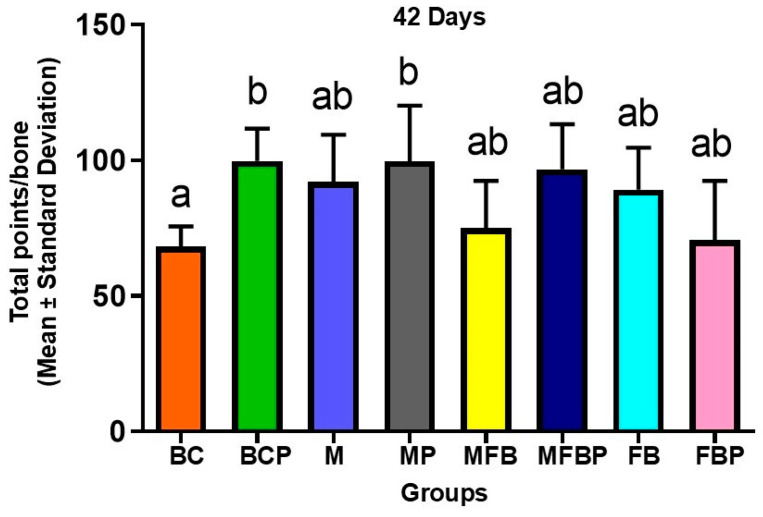
Histomorphometric analysis of newly formed bone volume at 42 days. Graph representing the mean and standard deviation of the total number of points over newly formed bone tissue per bone defect. Different letters (a, b, ab) indicate statistically significant differences between groups (*p* < 0.05). Groups: BC = Blood Clot; BCP = Blood Clot + PBM; M = Matrix; MP = Matrix + PBM; MFB = Matrix + FB; MFBP = Matrix + FB + PBM; FB = Fibrin Biopolymer; FBP = Fibrin Biopolymer + PBM.

The histomorphometric results of the means and standard deviations of the densities of the area occupied by new bone tissue formed (%), throughout the extension of the underlying monocortical and medullary lesion, were calculated using the point counting technique with the aid of planimetry (Table 2 and Figure 8). The data in the table revealed a statistically significant difference between BC (68.33 ± 7.394) compared to BCP and MP (99.83 ± 11.87 and 99.67 ± 20.58, respectively), but the other experimental groups presented *p* ≥ 0.05.

In relation to the same graft material treatment, with or without photobiomodulation therapy, the means were higher for the groups with photobiomodulation (BC vs. BCP, M vs. MP, MFB vs. MFBP), except between FB vs. FBP (89.17 ± 15.48 vs. 70.50 ± 21.88, respectively).

### 3.3. Analysis of the Birefringence of Collagen Fibers in the Bone Defect

**Figure 9:**
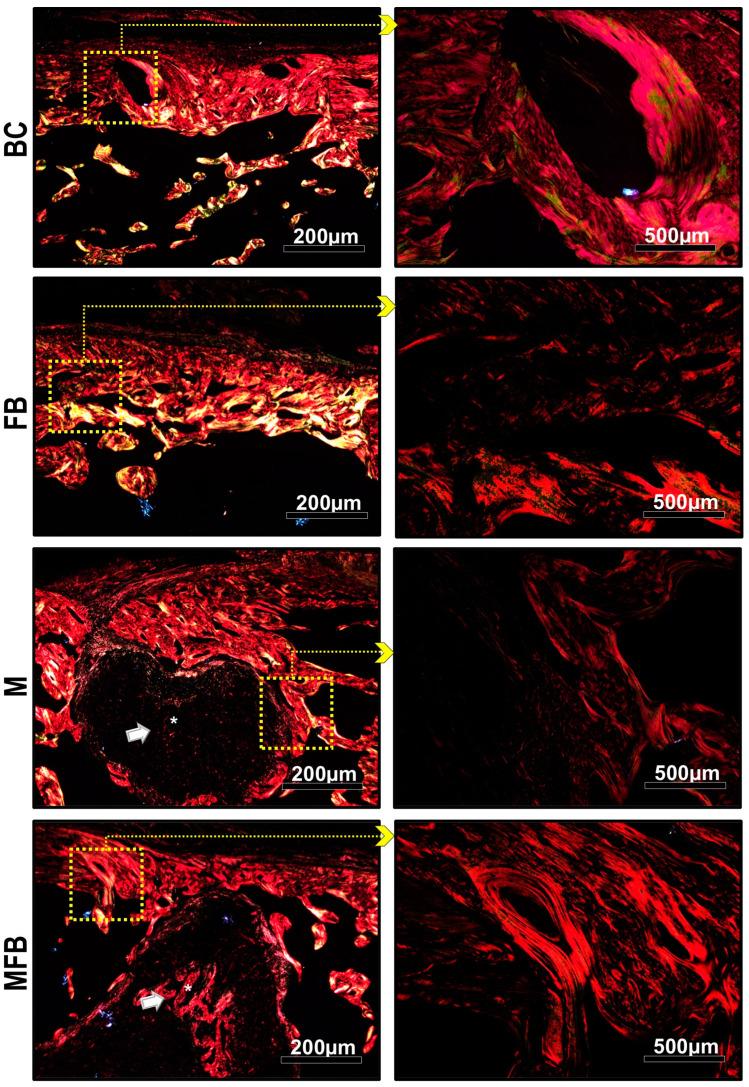
Histological images captured by polarized light microscopy of tibial bone defects, without low-level laser photobiomodulation, stained in picrosirius-red. Blood Clot (BC), Fibrin Biopolymer (FB), Matrix (M), Matrix + FB (MFB). Graft material (white arrow-asterisk). We used 5× objectives, with insets magnified at 20×; the scale bars were 200 µm and 500 µm, respectively.

**Figure 10:**
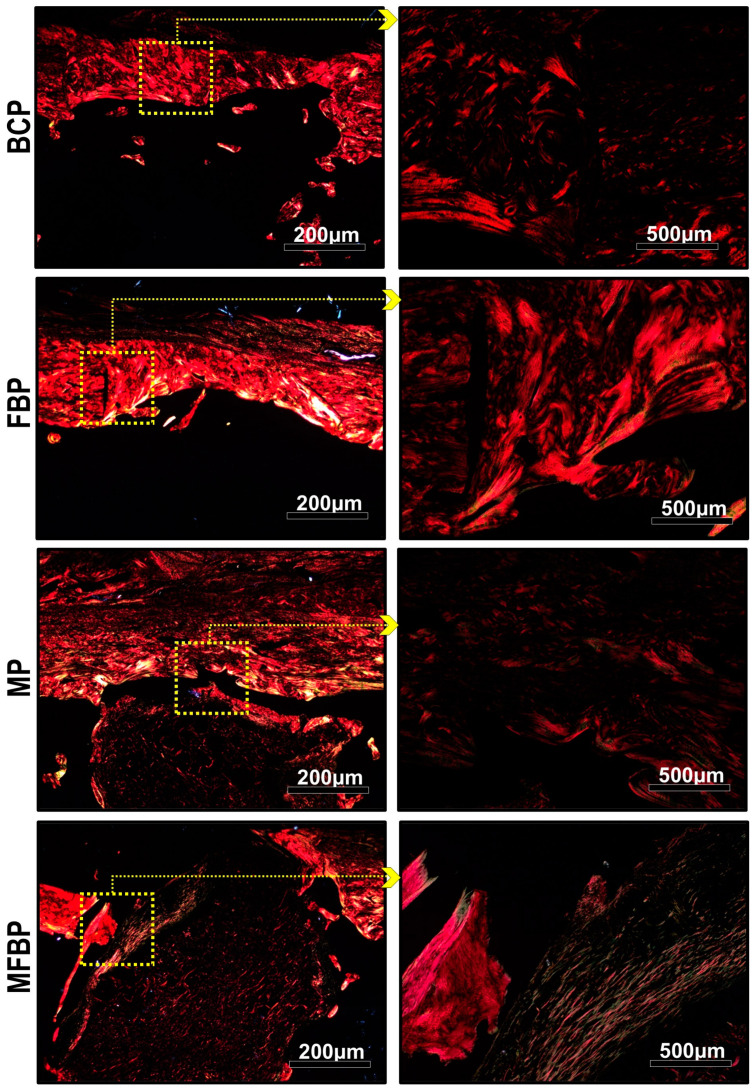
Histological images captured by polarized light microscopy of tibial bone defects, with low-level laser photobiomodulation, stained in picrosirius-red. Blood Clot + PBM (BCP), Fibrin Biopolymer + PBM (FBP), Matrix + PBM (MP), Matrix + FB + PBM (MFBP). We used 5× objectives, with insets magnified at 20×; the scale bars were 200 µm and 500 µm, respectively.

The images of histological sections stained using the picrosirius-red method for polarized light microscopy are shown in Figure 9 and Figure 10. The histochemical analysis of collagen fibers allows us to investigate the organization and maturation phase of the collagen fibrils of the newly formed bone matrix, which is directly proportional to the intensity of the birefringence brightness.

To analyze the degree of maturation and packaging of the collagen chains, different birefringences (green, yellow, and red) were identified in the injured areas. The thickest and most anisotropic collagen fibers present birefringence in shades of orange to red, and the thinnest and most disorganized ones, shades of yellow to green. In the groups treated with photobiomodulation (BCP, MP, FBP, and MFBP), qualitative evidence was observed in the appearance of the organization and maturation of collagen fibers, consistent with the predominance of orange-red birefringence and an insignificant amount of collagen fibers, emitting birefringence in the green spectrum, that is, suggesting the presence of immature and thinner fibers.

The images of groups M, MFB, MP, and MFBP showed the presence of graft fragments of the scaffolds in the injured areas with orange-red birefringence, confirming their presence in the conventional microscopy images, visualized in HE and Masson’s trichrome.

## 4. Discussion

This unprecedented preclinical study aimed to evaluate the biological behavior (in vivo) of two scaffolds, isolated or combined, in the tibia of rats: a porcine serous collagen matrix with nanohydroxyapatite and a heterologous fibrin biopolymer purified from snake venom. In addition, a photobiomodulation protocol was used to test its biostimulatory effects on the microenvironment of the surgically created bone defect, which improved the process of new bone formation.

The adaptive capacity of bone tissue is based on the balance between bone modeling and remodeling, in response to the need for adjustments to changes in the environment. However, excessive repairs pose a risk to bone homeostasis by destabilizing the apposition and reabsorption processes, potentially establishing a degenerative cycle between damage and repair, which may culminate in microdamage and loss of tissue functionality [44]. Despite the great advances in tissue engineering, the translation of innovative strategies for bone regeneration with clinical applicability still requires a long period of intense research [44].

In view of this, tissue bioengineering and regenerative medicine play a key role in developing and improving materials capable of preventing or even reducing this situation, providing a positive net balance between modeling and remodeling, and thus returning to native tissue [45]. The incorporation of electromagnetic therapies, such as low-power laser, has shown interesting results in bone tissue regeneration, but complex regulatory barriers are still observed regarding dosimetric parameters, and when associated with bone grafts [46].

Therefore, our group of researchers has highlighted the need to develop therapies with possible synergistic effects, given the lack of fully satisfactory and relevant results in the scientific literature in the morphofunctional restoration of bone tissue [26,47]. The biomaterials evaluated in this study were designed to design a three-dimensional microarchitecture capable of eliciting specific cellular responses mediated by cell signaling and regulatory factors within this new environment and corresponding to the mechanical behavior of the target tissue. Thus, they hypothetically serve as models for cell growth and may provide possible control over the cellular microenvironment, manipulating the repair process. In this way, they mimic the native structure and can be called biomimetic scaffolds [48,49,50].

However, it is necessary to point out that no material is endowed with all the desirable characteristics that enable successful bone consolidation [51,52]. This awakened in our group of researchers the need to evaluate associations of three-dimensional grafts with electromagnetic light therapy and to reestablish a microenvironment that better recapitulates the function of the native tissue, one that is a scaffold recognized by the recipient innate cells as its own, avoiding a foreign body-type immune response and fibrous encapsulation [53]. We tested a laser photobiomodulation protocol in the repair of long bones in rats that we used in previous studies [27,54], filled with the innovative biomimetic collagen matrix with nanohydroxyapatite, associated or not with a fibrin derivative, the only 100% heterologous fibrin biopolymer in the world [32].

Considering the studies on the impacts of electromagnetic radiation from laser therapy on cells with functional deficits, the activation of cytochrome C oxidase increases mitochondrial production of adenosine triphosphate (ATP), which in turn increases their metabolic activity [55,56]. At the same time, regulation of the reduction–oxidation (redox) state of the intracellular microenvironment can favor the expression of genes associated with tissue regeneration, repair, and immunological modulation, which ensure a coordinated regenerative effort [57,58].

Adopting the theory of principles and parameters of lasers, it is assumed that the photochemical and photophysical properties of some wavelengths are primarily responsible for the tissue response [59,60]. This is because this parameter is directly related to the resulting polarization phenomenon, which allows light to be absorbed by deep tissues at intensity thresholds sufficient to initiate desirable biochemical cascades. Thus, we can justify the use of an infrared laser with a wavelength of 808 nm in this study, as it is included in a spectral range of greater absorbance by bone chromophores [61,62,63,64].

However, it is worth noting that the physiological potential of photobiomodulation cannot be harnessed solely by light at a specific wavelength. Red and/or infrared light can promote the proliferation of several types of cells. Therefore, our selection of the protocol was detailed in the controllable variables of laser light, such as radiance, energy density/fluence, total energy, and spot area, as they provide favorable conditions for cell recruitment, active biomolecules, and synthesis of growth factors in situ, as observed in our previous studies and those of other researchers [22,65,66,67,68,69,70].

Previous studies corroborate our findings on the positive effects of photobiomodulation and biomimetic scaffolds in bone regeneration. Sadeghian and collaborators [71] reviewed the application of photobiomodulation therapy (PBM) in bone regeneration and found that, despite conflicting results, PBM showed positive effects on bone reconstruction in several clinical studies. Freitas et al. [66] highlighted that PBM accelerates bone regeneration by increasing vascularization and the inflammatory response, with a significant increase in osteocytes in the irradiated bone. De Marco et al. [72] demonstrated that PBM improves cell proliferation and matrix synthesis, corroborating our findings that photobiomodulation has a modulatory role in bone repair.

Additionally, Zhang et al. [73] reviewed the use of three-dimensional biomimetic scaffolds in tissue repair, highlighting that these scaffolds provide stable mechanical support and a favorable environment for cell infiltration and new tissue formation. Reference [29] emphasized the importance of biocompatibility and microporous structure in promoting bone growth, aligning with our results on the efficacy of collagen/nanohydroxyapatite scaffolds.

Our results indicate that photobiomodulation with an 808 nm infrared laser promoted bone formation and collagen fiber maturation, which is consistent with the findings of Sadeghian et al. [71] and Freitas et al. [66], who reported positive effects of PBMT on bone regeneration and the inflammatory response. The efficacy of collagen/nanohydroxyapatite scaffolds in our study is corroborated by the findings of [73] and Garcia et al. [29], who highlighted the importance of three-dimensional structure and biocompatibility in promoting bone regeneration.

Through these physiological phenomena caused by the laser to the damaged bone tissue, we transpose its applicability to defects grafted by materials with configurational and conformational microstructures compatible with native bone. Another point to be resolved is the functional and technical justification for the use of this combination of biomimetic scaffolds, which results from a synergistic interaction, which confers multiple benefits to the repair.

This includes the elasticity induced by the collagen matrix, allowing adequate contraction, protection against the collapse of soft tissues into the surgical area and cellular osteoconduction by nanohydroxyapatite particles and, finally, the biological functionality of the fibrin biopolymer mediated by cells, these fundamental attributes can recapitulate the functions of native bone tissue [33,74,75,76].

Prior to the evaluation of the grafts in vivo, this experimental protocol proposed qualitative two/three-dimensional microtomographic and histological analyses of the biomaterials of interest. Interestingly, grayscale and histological sections revealed the absence of nanohydroxyapatite particles, as well as mononuclear inflammatory foci or foreign body-type granulomatous reaction in the cortical area or within the bone trabeculae. This indicates the effectiveness of the grafting materials used, since the presence of different antigens in a graft favors the antigen–antibody reaction, which may compromise its degradation and compatibility. Sharing this statement, researchers emphasize that grafts that present immunogenic potential with cellular debris have reduced clinical application [77,78].

Microtomographically, the influence of infrared laser light was visible in the cortical interposition between the native bone and the newly formed bone, attributing to the intermingling of gray tones (see Figure 2 and Figure 3), more expressively in the three-dimensionally reconstructed slices [62]. These data are corroborated in the histological images by HE (see Figure 6 and Figure 7), which can be attributed to the speed of the initial phase of the healing process, most commonly inflammation and tissue debridement, culminating in a more advanced bone repair [79,80].

Masson’s trichrome staining (see Figure 4 and Figure 5) of the specimens revealed the presence of a vast collagenous matrix in all experimental groups, with a more oriented and thicker spatial conformation in the groups treated with photobiomodulation [5]. The strong contrast between the bluish collagen fibers and the reddish coloration of the cells allowed a clear identification of the osteoblastic cells, osteocytes and the formation of the osteoid matrix with mineralized areas [81,82,83].

Collectively, the histomorphometric data indicate substantially higher values in the BCP, MP and MFBP groups compared to the others. Exposure to infrared laser light demonstrated a modulating role in bone repair with obvious anabolic effects at the site of the bone defect. However, no significant difference was observed in these metric parameters between them [28,54,70,74].

Without further experimentation, we can only speculate that the combination of the collagen/nanohydroxyapatite matrix and fibrin biopolymer scaffolds altered porosity parameters, such as pore size distribution and interconnectivity, and did not accelerate bone growth compared to laser-treated defects [84,85,86,87]. However, this in vivo study demonstrated that these scaffolds promote and facilitate tissue remodeling, as evidenced by the recognition of the biological structure and the binding and bioadhesive behavior of the fibrin biopolymer.

Although not yet fully elucidated, all the factors responsible for the better performance in the dynamics of these tibial repairs, the high level of qualitative significance of the data obtained, indicate that the combination of these therapies is viable and has great potential for impact on clinical applicability aimed at effectively improving the reestablishment of compromised bone tissue.

It is important to recognize that the expected criteria for tissue bioengineering constructions are not confined to a single parameter. The observed outcomes from the hybrid combination of scaffolds have demonstrated improved quality of osteoregeneration and favorable conditions for bone consolidation. This improvement is evidenced by the alignment, orientation, and density of collagen fibers, as provided by oriented anisotropic birefringence [88,89,90,91,92].

Considering potential methodological limitations, our research team will continue the investigations to transition this preclinical study from the laboratory to clinical studies. Future applications in bone tissue engineering hold significant importance in Medicine, particularly Orthopedics, and Dentistry, specifically Periodontics and Bucomaxillofacial Surgery. The fibrin biopolymer is anticipated to undergo a clinical phase for chronic venous ulcer repair, currently in phase III at ANVISA (National Health Surveillance Agency, Brazil).

Although there remains a scarcity of research on the use of this combination of therapies in long bone repair dynamics, current findings offer a technical foundation for developing new methodological strategies. This could involve synthesizing the collagen/nanohydroxyapatite matrix and fibrin biopolymer scaffolds under controlled conditions tailored to the nature of the injury and the recipient tissue. Additionally, the innovative application of simultaneous red and infrared light [26] may enhance outcomes when used with the same matrices as those employed in this study.

Furthermore, considering the role of surface properties in nanoparticle bioactivity, future studies should also focus on assessing the zeta potential of nanohydroxyapatite. The literature suggests that negative zeta potentials enhance cellular interactions and protein adsorption, which are critical for bone regeneration. A more comprehensive characterization of this parameter could provide deeper information into the physicochemical behavior of the material and its influence on cellular responses.

## 5. Conclusions

In this preclinical study, we evaluated the efficacy of 808 nm infrared laser photobiomodulation (PBM) in the repair of long bones using biomimetic collagen matrices with nanohydroxyapatite and heterologous fibrin biopolymer. The study aimed to investigate whether the combination of these scaffolds with PBM could enhance bone regeneration in surgically created tibial defects in rats.

Our findings demonstrated that PBM significantly improved the bone healing process. The groups treated with PBM (BCP, MP, MFBP) showed higher averages of new bone formation compared to the control groups. Specifically, the BCP group had an average of 99.83 ± 11.87, the MP group had an average of 99.67 ± 20.58, and the MFBP group had an average of 96.67 ± 16.65 new bone formation. These results support the working thesis that PBM, in combination with biomimetic scaffolds, can accelerate and enhance bone repair.

The study also highlighted the potential of these biomimetic matrices to provide a favorable microenvironment for bone regeneration. The collagen matrix with nanohydroxyapatite offered structural support, while the fibrin biopolymer facilitated cellular adhesion and proliferation. The synergistic effect of these materials, combined with the biostimulatory properties of PBM, resulted in improved bone formation and maturation.

While these findings are promising, it is important to acknowledge the limitations of this study. The lack of long-term follow-up and potential variability in PBM application are factors that need to be addressed in future research. Additionally, further studies, including clinical trials, are necessary to translate these findings to human applications.

These findings suggest that the use of PBM in conjunction with biomimetic scaffolds could be a promising strategy for enhancing bone repair in clinical settings. Future research should focus on optimizing the parameters of PBM and further exploring the potential applications of these biomaterials in orthopedic and dental treatments. The results of this study provide a foundation for developing new therapeutic approaches that could reduce treatment time and costs, ultimately improving patient outcomes in bone regeneration.

## Figures and Tables

**Table 1:** Photobiomodulation protocol using low-level laser used in this study.

	Dosimetric Parameters
Wavelength	808 ± 10 nm
Operating mode	Continuous
Spectrum	GaAlAs
Useful power of the emitter	100 mW ± 20%
Beam area	0.043 cm^2^
Exposure time/point	60 s
Irradiance/power density	2.32 W/cm^2^
Energy density	139.53 J/cm^2^ per point
Number of points radiated	2 points
Application locations	Perpendicular defect surface
Application technique	2 points on the surface of the defect perpendicular in a clockwise direction (12 h/6 h)
Total energy	12 J
Number of sessions and frequency	Intraoperatively, remaining twice a week until euthanasia at 42 days

nm: nanometers; GaAlAs: gallium aluminum arsenide; mW: milliwatts; cm^2^: square centimeters; s: seconds; W: watts; J: joules.

**Table 2:** Means and standard deviations of the densities (%) of the area occupied by new bone tissue formed, in 42 days. Blood Clot (BC), Blood Clot + PBM (BCP), Matrix (M), Matrix + PBM (MP), Matrix + FB (MFB), Matrix + FB + PBM (MFBP), Fibrin Biopolymer (FB), Fibrin Biopolymer + PBM (FBP).

Groups 42 Days	BC	BCP	M	MP	MFB	MFBP	FB	FBP
Total points/bone (Mean ± Standard Deviation)	68.33 ± 7.394 ^a^	99.83 ± 11.87 ^b^	92.00 ± 17.47 ^ab^	99.67 ± 20.58 ^b^	75.00 ± 17.48 ^ab^	96.67 ± 16.65 ^ab^	89.17 ± 15.48 ^ab^	70.50 ± 21.88 ^ab^

Different lowercase letters (a ≠ b ≠ ab) indicate significant difference between groups, in the period of 42 days. ANOVA with Tukey’s post-test (*p* ≤ 0.05).

## Data Availability

The original contributions presented in this study are included in the article/Supplementary Materials. Further inquiries can be directed to the corresponding author.

## Supplementary Materials

The following supporting information can be downloaded at https://www.mdpi.com/article/10.3390/ma18081704/s1. Figure S1: Illustration of the methodology used to quantify bone volume (in %) in the cortical and medullary area of the defect.
