# Supplementary figures and images for: Efficacy of Three-Dimensional Bioactive Composites in Long Bone Repair with Photobiomodulation
Source: Materials (Basel). 2025 Apr 9;18(8):1704. doi: 10.3390/ma18081704 (PMC12028516; doi:10.3390/ma18081704)

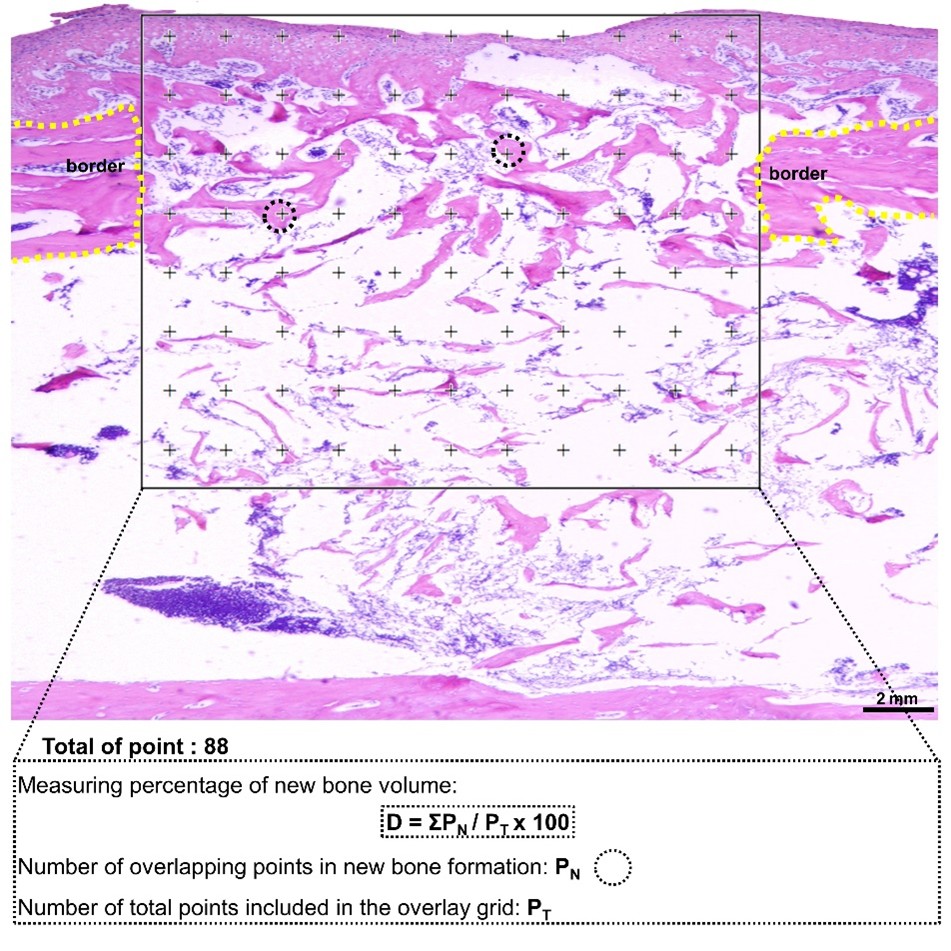

Supplement: Supplementary file 1 [file materials-18-01704-s001.zip › materials-3458902-supplementary/Figure S1.jpg]
